# 

**Published:** 1881-10

**Authors:** 


### CONTENTS.

### COMMUNICATIONS.

Antiseptic Treatment of Alveolar Abscesses........399
Physical Changes Affecting the Teeth of the Young........411
Extracting........415
The Relation of Dentistry to Medicine........422
Translations........424
An Interesting Case........425
Carbolic Acid Poisoning........426

### SELECTIONS.

Maternal Impressions........427
On the Pathology of Acute Periostitis........428
Spasm of the Œsophagus........429
The Committee on American Medical Colleges........430
What Teachers to Avoid........431
Solution and Suspension........432
Dentition and Misnomer........433
Microscopy and Pathology........435
New Method of Preparing the Spinal Cord for Microscopic Sections.. 436
Successful Transplantation of Human Bone........437
Reflex Neuralgia by Foreign Body in the Ear........437
New Injection Method........438

### EDITORIAL.

A Novel Case........438
The International Medical Congress........440
A Directory........442
Dissolution........442

